# Autologous Bone Marrow-Derived Chondrocytes for Patients with Knee Osteoarthritis: A Randomized Controlled Trial

**DOI:** 10.1155/2021/2146722

**Published:** 2021-11-20

**Authors:** Mir Sadat-Ali, Abdallah S. AlOmran, Sulaiman A. AlMousa, Hasan N. AlSayed, Khalid W. AlTabash, Mohammed Q. Azam, Tarek M. Hegazi, Sadananda Acharya

**Affiliations:** ^1^Department of Orthopaedic Surgery, Imam AbdulRahman Bin Faisal University, Dammam, Saudi Arabia; ^2^King Fahd Hospital of the University, AlKhobar, Saudi Arabia; ^3^Orthopaedic Surgery, All India Institute of Medical Sciences, Rishikesh, India; ^4^Radiology, Imam AbdulRahman Bin Faisal University, Dammam, Saudi Arabia; ^5^College of Public Health, Imam AbdulRahman Bin Faisal University, Dammam, Saudi Arabia

## Abstract

**Results:**

There were a total of 60 patients who were followed up. Three patients in Group II were removed from the analysis as they underwent total knee arthroplasty (TKA). A notably significant improvement was noticed in the ABMDC group on all scores of VAS and MKSSSF with *P* < 0.0001. The control group continued to be dissatisfied with the treatment they were taking.

**Conclusions:**

This study reveals that a single injection of 5 million of ABMDC was efficient in reducing the symptoms, improving the functional score and betterment of QOL.

## 1. Introduction

Osteoarthritis of the knee (OAK) is usually due to aging process, and many factors influence the severity of the disease. The prevalence of OAK of the knee is unknown, but recently, it was reported that over 12.5% of the population over 45 years of age suffer from OAK. [1] The incidence of OAK in Saudi Arabian population was found to be up to 3.5% [2, 3], but recent reports indicate that the prevalence of clinical OAK increased many folds to 13% [4] and radiological OA of knee to 53% in males and 60.9% in females [5].

The OAK is traditionally managed using nonsteroidal anti-inflammatory drugs (NSAIDs), physical therapy, and nutritional supplements [6–12]. The response to such treatment is not always absolute and requires steroid and hyaluronic acid injections and arthroscopic joint washout with diverse degrees of pain relief [13, 14].

In the recent past, incidence of OAK has increased, but no new modes of management have been added apart from the symptomatic treatment with NSAIDs and physical therapy. OAK progresses slowly, leading to joint destruction and reduction in quality of life and eventually ending with total knee arthroplasty (TKA). The management of OAK by the way of arthroplasty is expensive and complicated. Complications of TKA include vascular and neural injury, instability, malalignment, stiffness, joint infection, tibiofemoral dislocation, wear and tear of the polyethylene insert, osteolysis, implant loosening, and revisions [15].

Cell-based therapies particularly mesenchymal stromal cells (MSCs) for repair of the damaged cartilage and relief of pain have been tried in experimental animals with excellent results. MSCs for repair of damaged cartilage and for relief was used in sheep model with good outcome [16]. Jo et al. [17] reported that after injecting MSCs into the osteoarthritic knee, follow-up at 3, 6, and 12 months showed improvement in function and total relief of pain of the knee joint without causing adverse events and reduced cartilage defects by regeneration of the cartilage, which was similar to hyaline cartilage histologically. Soler et al. [18] reported the outcome of autologous MSCs in patients with grades II and III of Kellgren–Lawrence grading and found that a single intra-articular injection of the MSCs was safe and gave complete pain relief with improved quality of life with signs of cartilage repair based on the MRI.

There is increasing prevalence of OAK among Saudi Arabian population with the number of total knee arthroplasty procedures on the rise even in young patients, and many patients are left to suffer and the disease progression occurs, when other alternatives are available. Keeping this in mind to find an alternate and safe treatment, we decided to perform this pilot study using ABMDC as a single intra-articular knee injection of 5 million cells and to compare it with the standard treatment in the same cohort population.

## 2. Methods and Patients

This is a prospective, open-label, single-dose, clinical trial of injection of chondrocytes of 10^5^ culture expanded cells. The study was approved by the Institutional Review Board of Imam AbdulRahman Bin Faisal University, Dammam, vide IRB-2018-01-314, dated 19/12/2018, and informed written consent was taken from all the patients before staring the treatment plan. All procedures in this study were conducted in accordance with the guidelines as laid down by the Institutional Review Board of Imam AbdulRahman Bin Faisal University, Dammam. The sample size was based on the ClinicalTrials.gov Identifier NCT03818737 (Multicenter Trial of Stem Cell Therapy for Osteoarthritis (MILES)) [19].

The objective of the study is to assess the benefits of autologous bone marrow-derived chondrocyte injection in patients with OAK and to assess safety, efficacy, and tolerability of this treatment. Efficacy was measured using the Visual Analogue Scale for pain, Short Knee Society score, pain at rest, pain on movement, satisfaction with daily life, and quality of life. The inclusion criteria were patients being between 45 and 70 years of age, diagnosed with OAK for at least 12 months based on American College of Rheumatology (ACR) criteria, and unsatisfied with conservative therapy of NSAIDs and physiotherapy. Patients should be able to communicate with the researchers and answer to the questionnaires, agreeing to participate and signing informed consent voluntarily.

The first 30 patients who agreed to be part of the chondrocyte treatment were part of Group I, and all second patients (30) who were being treated conservatively were Group II patients. The exclusion criteria were past knee intra-articular steroid/hyaluronic acid injection within the last 3 months, any knee surgery within the last 6 months, pregnancy and breast-feeding (women of childbearing age need the pregnancy test), mental disorders, and drug dependency. Patients were divided into two groups.

### 2.1. Bone Marrow Aspiration

Under general anesthesia, 20 cc of bone marrow was aspirated from the patients' iliac crest following standard procedures. The aspirate was added to a sterile bone marrow transplantation medium (Hanks' Balanced Salt Solution with 10,000 units/mL of penicillin, 10,000 *μ*g/mL of streptomycin, and 25 *μ*g/mL of amphotericin B). The samples were properly labeled and were immediately transported to the stem cell laboratory under ice-cold conditions.

### 2.2. MSC Separation and Expansion

Bone marrow in the transportation medium was overlaid on approximately 20 ml of sterile Ficoll-Paque Plus (Merck KGaA, Darmstadt, Germany) taken in a conical centrifuge tube (Sarstedt AG & Co, Nümbrecht, Germany) and centrifuged in a swing bucket centrifuge (Sorvall ST8, Thermo Fisher Scientific, Massachusetts, USA) at 2000 rpm for 5 minutes at 4°C. The resultant layer containing mononuclear cells were siphoned out and seeded onto a T75 tissue culture flask (Greiner Bio-One GmbH, Frickenhausen, Germany) at the rate of 5000 cells/cm [2]. Approximately 20 ml of the MSC culture medium (CTS™ StemPro™ MSC SFM, Thermo Fisher, Massachusetts, USA) was added, and the flask was incubated at 37 C, with 5% CO_2_ and 95% RH in a CO_2_ incubator. Adherent cells were cultured, and the process was continued with change of the medium every second day till cells became confluent. The cells were trypsinised, harvested, and used for chondrocyte differentiation. A portion of the cells was used for cell characterization using MSC specific antibodies in a flow cytometer (Human MSC Phenotyping Kit from Miltenyi Biotec (Auburn, CA, USA)). Positive expression of CD73, CD90, and CD105 and negative for CD45 were confirmed.

### 2.3. Chondrocyte Differentiation

The MSC cell aggregate was added to micro-mass culture using the MSC culture medium on a 90 mm cell culture dish (Nunc, Denmark). After two hours of adding the MSCs, the medium was washed off and fresh chondrocyte differentiation medium (CTS StemPro MSC medium supplemented with 10^−7^ M dexamethasone, 1 *μ*M ascorbate-2-phosphate, 1% sodium pyruvate, and 10 ng/ml transforming growth factor-beta 1 (TGF-*β*1), Thermo Fisher, Massachusetts, USA)) was added [20]. The micro-mass culture was allowed to differentiate for 21 days with medium changes for every third day. The cells were harvested at the end of the culture and were characterized for the expression of MMP13 using a flow cytometer.

#### 2.3.1. Intervention

Group I received ABMDC cell suspension which was prepared in 0.5 cc of sterile NSS at the rate of 1 × 10 [6] cells/body weight of the patient. The cell suspension was injected intra-articularly in the affected joint as a single-dose injection. Group I participants did not receive any further treatment including physical therapy.

**Figure 1:**
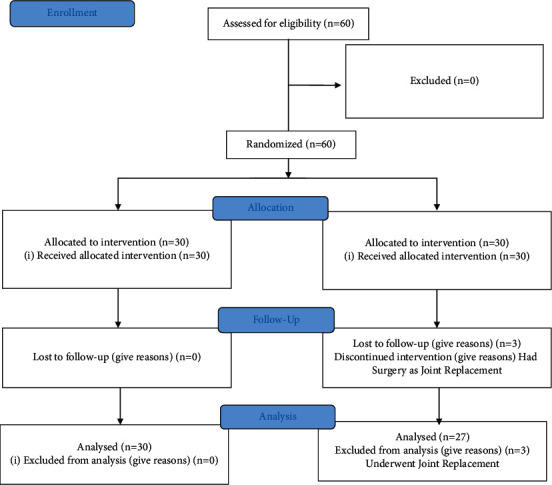
CONSORT flow diagram.

In Group II, we continued the NSAIDs and physical therapy as needed. Before the treatment, patients were meticulously examined and data were entered for age, sex, Body Mass Index (BMI), Visual Analogue Scale (VAS), Modified Knee Society Score-Short Form (KSSSF), and QOL. Patients were followed up for 3, 12, and 24 months.  Figure 1 gives the CONSORT flow chart.

#### 2.3.2. Statistical Analysis

The data was entered in the database and analyzed using SPSS (Statistical Package for the Social Sciences), version 25.0, Chicago, Illinois. A *P* value of <0.05 was considered statistically significant with a confidence interval (CI) of 95%.

## 3. Results

A total of 60 patients were included and followed up (Figure 1). Three patients in Group II were removed from the analysis as they underwent total knee arthroplasty. All patients were followed for 3, 12, and 24 months. In 30 patients (treatment group), a single ABMDC of 5 million cells was injected. A control group of 27 patients were treated with NSAIDs, physical therapy, and Durolane, a hyaluronic acid injection. The demographic data of patients in groups I and II were similar before the treatment was instituted.  Table 1 shows all the parameters assessed including age, KL grading, VAS, MKSSSF, QOL, daily living, and pain at movement with no statistically significant difference except for pain at rest which was significantly higher in group I at *P* value < 0.0009. There were no adverse effects in group I, but in group II, 1 patient (3.7%) had local cellulitis which was treated by oral antibiotics.

## 4. Clinical Efficacy

A robust improvement was observed in the ABMDC group on all scores of pain relief, VAS, QOL, MKSSSF, pain at rest, pain on movement, and assessment of daily life with a *P* value of <0.0001 for all parameters. The improvement was significant from 3 months onwards and continued for the period of follow-up of 24 months. The MKSSSF score at baseline was the control group that continued to suffer and be dissatisfied with the treatment (Table 2). The MKSSSF score at baseline in Group I was 37.40 ± 6.46 and consistently improved till the last follow-up at 24 months to 82.18 ± 3.79. The change from the baseline to the 1^st^ follow-up at 12 months was statistically significant at *P* < 0.0001, and between 12 months and 24 months, the *P* value was <0.0003. In Group II, at the start of the study, the Modified Knee Scoring Score Short Form was 39.34 ± 6.83; at 12 months, it was 42.31 ± 5.72, and at 24 months, it was 40.15 ± 5.88. At baseline and 12 months, *P* < 0.0892 and at 24 months, *P* < 0.6424.

## 5. Radiological Outcomes

**Figure 2:**
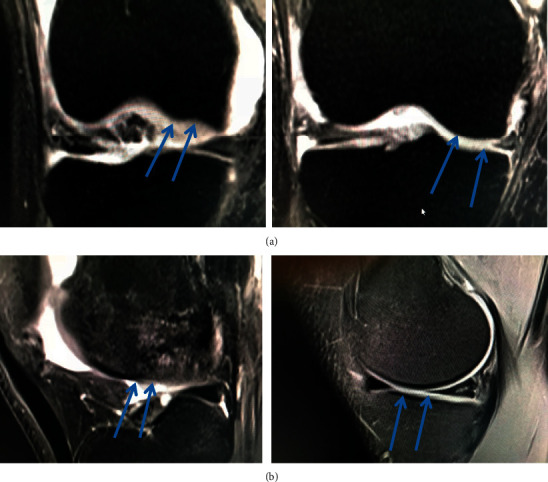
(a) AP view of the MRI of the knee joint showing depression in the femoral condyle (blue arrows) due to the damaged cartilage which completely recovered post injection of ABMDC. (b) Lateral view of the MRI of knee joint with erosion of the cartilage of the femoral condyle and post-injection regeneration of the damaged cartilage (blue arrows).

Kellgren–Lawrence grading and the assessment of the joint space width were not performed. The initial MRI examinations were studied and showed varying levels of cartilage defect, and a follow-up MRI showed healed cartilage defect. The regenerated cartilage appeared leveled and smoother (Figure 2).

## 6. Discussion

Our preliminary study showed that ABMDC intra-articular injection in KL II and III grade knees showed an excellent improvement compared to the patients in the standard treatment protocol. All of the three parameters tested in the ABMDC group, VAS, QOL, and MKSSSF, showed significant improvements compared to the control group over 24 months. We did not observe any apparent early or late adverse events due to the ABMDC intra-articular injection. Radiological improvement was noticed on evaluation by a follow-up MRI with evidence of the regenerated articular cartilage and disappearance of the depression of the cartilage as seen in the pretreatment MRI.

The initial report of Orozco and his colleagues [21] on clinical efficacy and safety of autologous human bone marrow-derived stem cells in OAK showed that there were a significant decrease in the knee pain and improved changes on the MRI scans. Vega et al. [22] and Gupta et al. [23] used allogenic bone marrow-derived stem cells and compared those with hyaluronic acid injections and reported functional improvement and no adverse events in the patients receiving stem cell injections. A recent systematic review and meta-analysis confirmed positive conclusions about clinical efficacy of MSCs to treat pain and decreased function in knee OA [24]. Park et al. [25] used a combination of composite of allogeneic umbilical cord blood-derived mesenchymal stem cells and hyaluronate hydrogel for cartilage regeneration in OA patients and reported a 7-year follow-up. They reported that the VAS and International Knee Documentation Committee scores improved at 6 months and that this improvement remained at 7 years of follow-up. The reported mechanism by which bone marrow stromal cells reduce the pain and regenerate the cartilage is by differentiating into chondrocytes or by conversion of the remaining prochondrocytes into mature chondrocytes [23]. The main function of the chondrocytes is to produce extracellular matrix and collagen I and II in the process of repairing the damaged cartilage. There is enough evidence that aging has a detrimental effect on stem cells in quality and quantity and hence self-healing is low [26].

In our study, we bypassed the route of mesenchymal stem cells and directly used 10^6^ million autologous chondrocytes and found that the clinical effect was observed within weeks and secondly there was improvement in all the tested parameters, which were found to be better than the conservative methods of treatment. We did not encounter any risks involved in using the autologous chondrocytes, and we felt patients were more serene in using their own cells rather than allogeneic material. Moreover, in an earlier study, we used ABMDC for healing of the torn meniscus with convincing results [27].

A year ago, osteoarthritis was reported to be the fourth leading cause of chronic disability in the world [28] and is still being treated by conservative methods using nonsteroidal anti-inflammatory drugs (NSAIDs) and intra-articular injections. All the treatments have their limitations and long-term complications [29–31]. Even though our results and other meticulous analysis show that the overall results of the MSCs/chondrocyte treatment of OAK in both efficacy and safety are steps ahead and far superior to the conservative management, still this type of treatment is seen with skepticism and cynicism even among the treating physicians.

The limitations of our study are small size and absence of pretreatment and posttreatment arthroscopy to assess the cartilage repair, with the strength of the study being a matched group with three different parameters compared. In conclusion, our randomized control study persuades us to state that ABMDC is a better mode and option of treatment of OAK of KL grades II and III and it should be offered to the patients if the facilities exist to give such treatment.

## 7. Conclusions

Our study confirmed that a single injection of 5 million ABMDCs was efficient in reducing the symptoms and improving the functional score and betterment of QOL. We believe that such studies should be carried out nationwide to limit the progression of the OAK and improve the QOL of patients as they age.

## Figures and Tables

**Table 1:** Demographic data of baseline characteristics.

	ABMDC-group I	Conservative therapy-group II	*P* value
Age (years)	56.18 ± 6.56	56.75 ± 5.77	0.7302
Males	9	14	0.5
Females	24	16	0.2
KL grades II and III	18	16	0.2
Visual Analogue Scale^*∗*^	6.65 ± 0.09	6.96 ± 1.1	0.1294
Modified Knee Scoring Score-Short Form^*∗∗*^	37.40 ± 6.46	39.34 ± 6.83	0.2753
Pain at rest^*∗*^	3.46 ± 0.50	3.85 ± 0.6	0.009
Pain on movement^*∗*^	7.0 ± 0.87	7.15 ± 0.88	0.6682
Daily living^*∗*^	4.49 ± 0.75	4.34 ± 0.97	0.5141
Quality of life (QOL)^*∗*^	4.87 ± 1.09	5.25 ± 1.13	0.0716

^*∗*^Score out of 10 and ^*∗∗*^score out of 100.

**Table 2:** Comparison between groups at 3, 12, and 24 months.

Parameters	ABMDC-group I	Conservative therapy-group II	*P* value (95% CI)
VAS at 3 months	3.09 ± 0.64	6.75 ± 0.84	0.0001 (3.2659 to 4.0541)
MKSSSF at 3 months	57.48 ± 8.18	38.87 ± 6.33	0.0001 (−22.5247 to −14.6953)
Pain without movement at 3 months	3.37 ± 0.49	4.34 ± 0.6	0.0001 (0.6804 to 1.2596)
Pain on movement at 3 months	3.65 ± 0.60	7.09 ± 0.73	0.0001 (3.0867 to 3.7933)
Daily living at 3 months	5.28 ± 1.02	4.59 ± 0.75	0.0056 (−1.1698 to −0.2102)
QOL at 3 months	7.9 ± 0.64	4.81 ± 0.89	0.0001 (−3.4985 to −2.6815)
VAS at 12 months	2.59 ± 0.06	6.71 ± 0.72	0.0001 (3.8558 to 4.3842)
MKSSSF at 12 months	72.68 ± 13.15	42.31 ± 5.72	0.0001 (−35.860 to −24.880)
Pain without movement at 12 months	2.43 ± 0.50	4.5 ± 0.8	0.0001 (1.7196 to 2.4204)
Pain on movement at 12 months	2.47 ± 0.55	7.06 ± 0.71	0.0001 (4.2547 to 4.9253)
Daily living at 12 months	5.625 ± 1.21	4.75 ± 0.76	0.0021 (−1.4185 to −0.3315)
QOL at 12 months	8.175 ± 0.59	4.81 ± 0.89	0.0001 (−3.7621 to −2.9679)
VAS at 24 months	1.87 ± 0.42	6.34 ± 0.70	0.0001 (4.1671 to 4.7729)
MKSSSF at 24 months	82.18 ± 3.79	40.15 ± 5.88	0.0001 (−44.630 to −39.430)
Pain without movement at 24 months	1.75 ± 0.43	4.81 ± 0.64	0.0001 (2.7732 to 3.3468)
Pain on movement at 24 months	1.91 ± 0.39	6.9 ± 0.58	0.0001 (4.7300 to 5.2500)
Daily living at 24 months	7.4 ± 0.79	5.03 ± 0.82	0.0001 (−2.7976 to −1.9424)
QOL at 24 months	8.5 ± 0.50	4.34 ± 0.86	0.0001 (−4.5289 to −3.7911)

## Data Availability

Raw data were generated at King Fahd Hospital of the University, AlKhobar, Saudi Arabia. Derived data supporting the findings of this study are available from the corresponding author on request at dsr@iau.edu.sa.

## References

[1] Jordan K., Croft P. (2005). The prevalence and history of knee osteoarthritis in general practice: a case–control study. *Family Practice*.

[2] Sadat-Ali M., Al-Gindan Y., Al-Mousa M., Al-Rubaish A., Al-Omari E. (1996). Osteoarthritis of the knee among Saudi Arabian security forces personnel. *Military Medicine*.

[3] Ahlberg A., Linder B., Binhemd T. A. (1990). Osteoarthritis of the hip and knee in Saudi Arabia. *International Orthopaedics*.

[4] Al-Arfaj A. S., Alballa S. R., Al-Saleh S. S. (2003). Knee osteoarthritis in Al-Qaseem, Saudi Arabia. *Saudi Medical Journal*.

[5] Al-Arfaj A., Al-Boukai A. A. (2002). Prevalence of radiographic knee osteoarthritis in Saudi Arabia. *Clinical Rheumatology*.

[6] Persson M. S. M., Stocks J., Varadi G. (2020). Predicting response to topical non-steroidal anti-inflammatory drugs in osteoarthritis: an individual patient data meta-analysis of randomized controlled trials. *Rheumatology*.

[7] Wolff D. G., Christophersen C., Brown S. M., Mulcahey M. K. (2021). Topical nonsteroidal anti-inflammatory drugs in the treatment of knee osteoarthritis: a systematic review and meta-analysis. *The Physician and Sportsmedicine*.

[8] Zeng C., Doherty M., Persson M. S. M. (2021). Comparative efficacy and safety of acetaminophen, topical and oral non-steroidal anti-inflammatory drugs for knee osteoarthritis: evidence from a network meta-analysis of randomized controlled trials and real-world data. *Osteoarthritis and Cartilage*.

[9] Kakatum N., Pinsornsak P., Kanokkangsadal P., Ooraikul B., Itharat A. (2021). Efficacy and safety of sahastara remedy extract capsule in primary knee osteoarthritis: a randomized double-blinded active-controlled trial. *Evidence-based Complementary and Alternative Medicine*.

[10] Askari A., Ravansalar S. A., Naghizadeh M. M. (2019). The efficacy of topical sesame oil in patients with knee osteoarthritis: a randomized double-blinded active-controlled non-inferiority clinical trial. *Complementary Therapies in Medicine*.

[11] Anvari M., Dortaj H., Hashemibeni B., Pourentezari M. (2020). Application of some herbal medicine used for the treatment of osteoarthritis and chondrogenesis. *Iranian Traditional Medicine*.

[12] Sadat-Ali M., Al-Habdan I., El-Hassan A. Y. (2006). ? is there an alternative to NSAIDs and cox-2 inhibitors in the management of osteoarthritis of knee. *Ostetoporosis International*.

[13] Chevalier X. (2010). Intra-articular treatments for osteoarthritis: new perspectives. *Current Drug Targets*.

[14] Kirkley A., Birmingham T., Litchfield R. (2009). Arthroscopic surgery did not provide additional benefit to physical and benefit to physical and medical therapy for osteoarthritis of the knee. *Journal of Bone and Joint Surgery American Volume*.

[15] Healy W. L., Della Valle C. J., Iorio R. (2013). Complications of total knee arthroplasty standardized list and definitions of the knee society. *Clinical Orthopaedics and Related Research*.

[16] Caminal M., Fonseca C., Peris D. (2014). Use of a chronic model of articular cartilage and meniscal injury for the assessment of long-term effects after autologous mesenchymal stromal cell treatment in sheep. *New Biotech*.

[17] Jo C. H., Lee Y. G., Shin W. H. (2014). Intra-articular injection of mesenchymal stem cells for the treatment of osteoarthritis of the knee: a proof-of-concept clinical trial. *Stem Cells*.

[18] Soler R., Orozco L., Munar A. (2016). Final results of a phase I-II trial using ex vivo expanded autologous mesenchymal stromal cells for the treatment of osteoarthritis of the knee confirming safety and suggesting cartilage regeneration. *The Knee*.

[19] (2021). Multicenter trial of stem cell therapy for osteoarthritis (MILES). https://www.clinicaltrials.gov.

[20] Solchaga L. A., Penick K. J., Welter J. F. (2011). Chondrogenic differentiation of bone marrow-derived mesenchymal stem cells: tips and tricks. *Mesenchymal Stem Cell Assays and Applications*.

[21] Orozco L., Munar A., Soler R. (2013). Treatment of knee osteoarthritis with autologous mesenchymal stem cells: a pilot study. *Transplantation*.

[22] Vega A., Martín-Ferrero M. A., Del Canto F. (2015). Treatment of knee osteoarthritis with allogeneic bone marrow mesenchymal stem cells: a random-ized controlled trial. *Transplantation*.

[23] Gupta P. K., Chullikana A., Rengasamy M. (2016). Efficacy and safety of adult human bone marrow-derived, cultured, pooled, allogeneic mesenchymal stromal cells (Stempeucel^®^): preclinical and clinical trial in osteoarthritis of the knee joint. *Arthritis Research & Therapy*.

[24] Xia P., Wang X., Lin Q., Li X. (2015). Efficacy of mesenchymal stem cells injection for the management of knee osteoarthritis: a systematic review and meta-analysis. *International Orthopaedics*.

[25] Park Y.-B., Ha C.-W., Lee C.-H., Yoon Y. C., Park Y.-G. (2017). Cartilage regeneration in osteoarthritic patients by a composite of allogeneic umbilical cord blood-derived mesenchymal stem cells and hyaluronate hydrogel: results from a clinical trial for safety and proof-of-concept with 7 Years of extended follow-up. *Stem Cells Translational Medicine*.

[26] Ahmed A. S. I., Sheng M. H., Wasnik S., Baylink D. J., Lau K.-H. W. (2017). Effect of aging on stem cells. *World Journal of Experimental Medicine*.

[27] N AlSayed H., Sadat-Ali M., Z Uddin F., M Alani F., Acharya S. (2018). Outcome of bone marrow derived chondrocyte injection for meniscal injuries: a preliminary study. *Trends in Medicine*.

[28] Hunter D. J., Bierma-Zeinstra S. (2019). Osteoarthritis. *The Lancet*.

[29] Caldwell B., Aldington S., Weatherall M., Shirtcliffe P., Beasley R. (2006). Risk of cardiovascular events and celecoxib: a systematic review and meta-analysis. *Journal of the Royal Society of Medicine*.

[30] Evans C. H., Kraus V. B., Setton L. A. (2014). Progress in intra-articular therapy. *Nature Reviews Rheumatology*.

[31] Campbell K. A., Erickson B. J., Saltzman B. M. (2015). Is local viscosupplementation injection clinically superior to other therapies in the treatment of osteoarthritis of the knee: a systematic re- view of overlapping meta-analyses. *Arthroscopy: The Journal of Arthroscopic & Related Surgery*.

